# Supplementary figures and images for: Transcriptomic analysis of Pacific white shrimp (Litopenaeus vannamei, Boone 1931) in response to acute hepatopancreatic necrosis disease caused by Vibrio parahaemolyticus
Source: PLoS One. 2019 Aug 13;14(8):e0220993. doi: 10.1371/journal.pone.0220993 (PMC6692014; doi:10.1371/journal.pone.0220993)

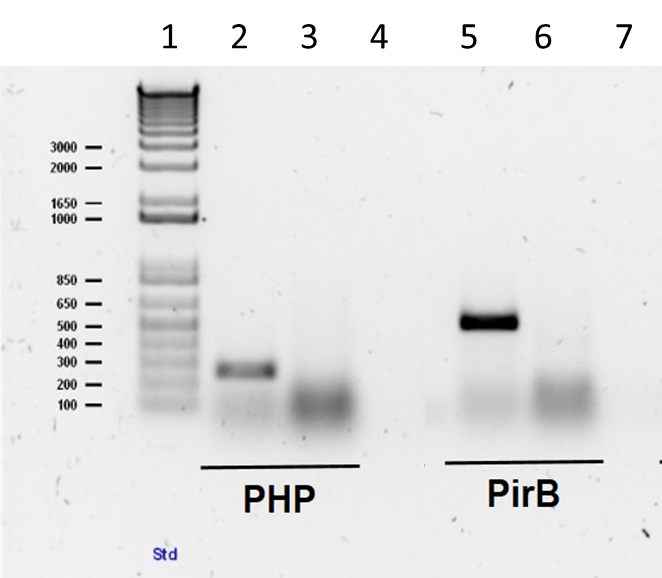

Supplement: S1 Fig — 2 and 5 lane shows Vibrio parahaemolyticus IPNGS16, 3 and 6 lane shows Vibrio parahaemolyticus CAIM170 (Non-AHPND). 4 and 7 lane shows negative control (water). Lane 1 shows molecular marker 1 kb plus. (TIF) [file pone.0220993.s001.tif]

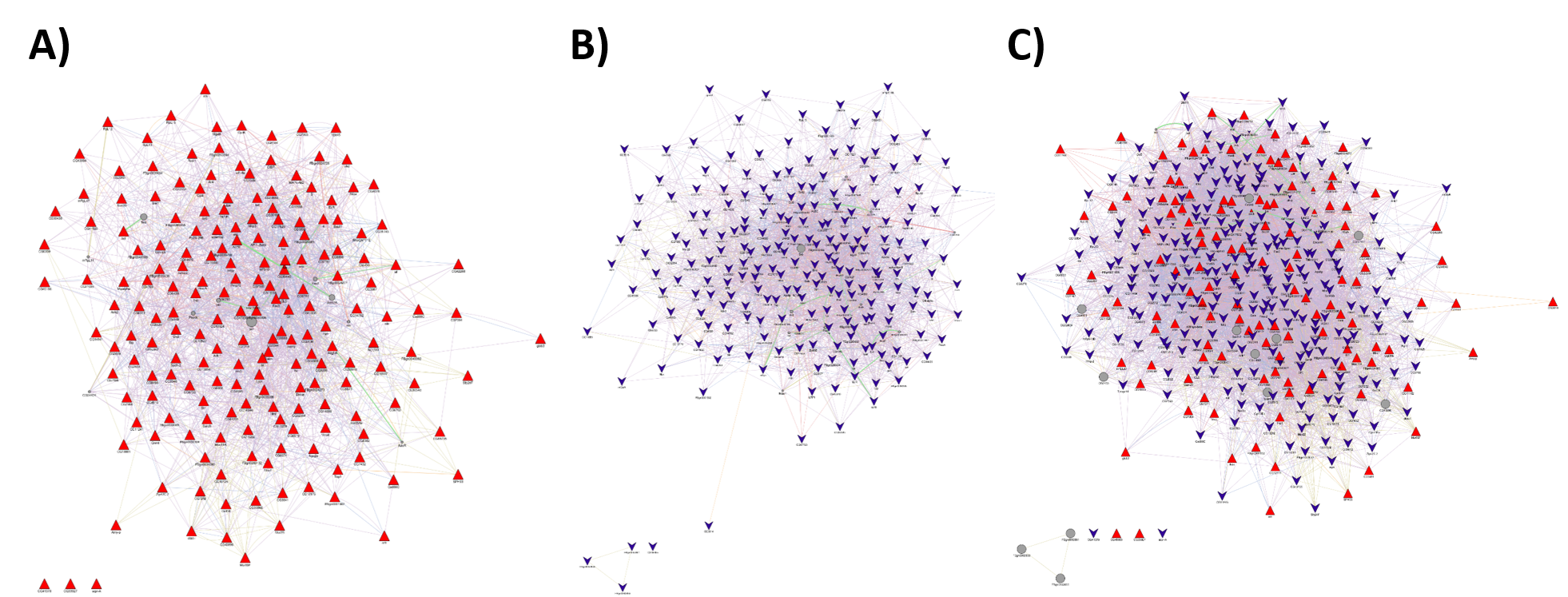

Supplement: S2 Fig — The Networks represent protein- protein interactions. A) Network 1 for up-regulated transcripts. B) Network 2 for down-regulated transcripts and C) Network 3 a combination of up and down-regulated transcripts. Red triangles represent up-regulated transcripts, blue inverted chevrons represent down-regulated transcripts, and gray circles represent GeneMANIA predicted proteins for each network. (TIF) [file pone.0220993.s002.tif]

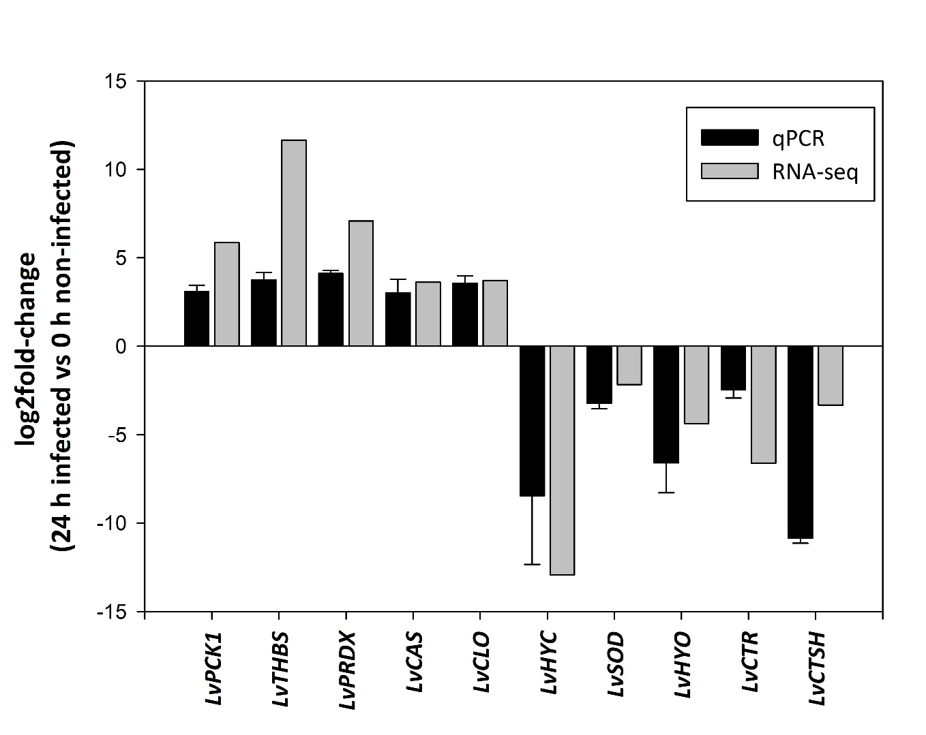

Supplement: S3 Fig — Time 0 h post-infection vs. 24 h post-infection. (TIF) [file pone.0220993.s003.tif]
