# Supplementary material for: Transcriptomic analysis of Pacific white shrimp (Litopenaeus vannamei, Boone 1931) in response to acute hepatopancreatic necrosis disease caused by Vibrio parahaemolyticus
Source: PLoS One. 2019 Aug 13;14(8):e0220993. doi: 10.1371/journal.pone.0220993 (PMC6692014; doi:10.1371/journal.pone.0220993)
Supplement: Attachment — Submitted filename: R2R.docx [file pone.0220993.s010.docx]

La Paz, Baja California Sur, Mexico, May 31^th^ 2019

**Professor Joerg Heber**

**Editor-in-Chief**

**PLOS ONE Journal**

Dear Professor Heber,

I am pleased to re-submit the manuscript entitled "Transcriptomic analysis of Pacific White Shrimp (*Litopenaeus vannamei*, Boone 1931) in response to acute hepatopancreatic necrosis disease caused by *Vibrio parahaemolyticus*" by Adrián E. Velázquez-Lizárraga, José Luis Juárez-Morales, Ilie S. Racotta, Humberto Villarreal-Colmenares, Oswaldo Valdes-Lopez5, Antonio Luna-González, Carmen Rodríguez-Jaramillo, Norma Estrada and Felipe Ascencio, to be reconsidered for publication in PLOS ONE.

The first time, the reviewers made very critical comments about the conclusions and the work experimental limitations to reach such conclusions. This time we restructured the manuscript and revised both, the scientific sense and the English language.

I consider that PLOS ONE journal has high visibility and accessibility in the world as well as its published works. We believe that this new version complies with the PLOS ONE standards, in this manuscript we are reporting the transcriptional response of *L. vannamei* in the early stages of *V. parahaemolyticus* infection.

We would like you to reconsider this new version of the manuscript, we placed the corrections and comments made by the reviewers at the end of this letter.

Sincerely yours

__________________________

**Dr. Felipe Ascencio**

[ascencio@cibnor.mx](mailto:ascencio@cibnor.mx)

| **REVIEWER 1** | |
| --- | --- |
| **Comments to the Author** | **Answer to reviewer** |
| A total of 10 transcripts were further selected to evaluate the relative expression with qPCR. Relative expression of the oxidation-reduction process, hemocyanin, clottable protein, thrombospondin increases the expression at 24 hours in an infected group. In conclusion, the authors suggest that the effect of hemocyanin and clottable protein have an effect in neutralizing PirABvp toxins. The approach formulated in this study of the disease can modify the poisoning effect of the toxins and the Vibrio parahaemolyticus infective process. Basically, this is only a transcriptome analysis without any functional study. At least, the speculation of these authors with “the effect of hemocyanin and clottable protein have an effect in neutralizing PirABvp toxins” should be biologically tested. Besides, the writing and format of this manuscript should be improved. | We have modified the focus of the discussion and have only analyzed and discussed the data obtained from the RNA-seq and the qPCR data. The writing, grammar and syntax of the manuscript was reviewed by an English editor. |
| **REVIEWER 2** | |
| **Comments to the Author** | **Answer to reviewer** |
| Acute hepatopancreatic necrosis disease (AHPND) emerged as a new disease of shrimp. In this manuscript, histopathological analysis on shrimp infected with V. parahaemolyticus with the plasmid that encodes PirABvp toxins was performed to explore the response of shrimp to AHPND. Furthermore, the author used RNA sequencing to compare the gene expression patterns between non-infected and infected shrimp. A significant amount of data was collected, but the analysis and discussion for the results were inadequate. Before submitting this manuscript, the concerns as follows should be addressed. | The manuscript was restructured in the discussion and in the conclusion based solely on the results obtained L541-678.. |
| If AHPND was caused by PirABvp toxins from the plasmid of V. parahaemolyticus, why did the author not use shrimp  infected Non-AHPND bacteria as the control such as Vibrio parahaemolyticus CAIM170 | The strain *V. parahaemolyticus* CAIM170 was isolated from shrimp with vibriosis, but currently it has no pathogenicity against the shrimp, and it was used as a negative control to detect the virulence genes of the AHPND. In addition, the method of infection used in this experiment was by immersion, emulating the natural conditions. |
| Why did the author choose samples at 24 hours for transcriptome sequencing? | We choose this time point because LC_50_ was calculated at 24hpi, where half of the organisms died with a dose of 660 CFU/ml. Therefore we were interested in studying the transcriptional response of the AHPND infected hepatopancreas at this time point. |
| New information to describe the response of shrimp to AHPND should be highlighted in this paper | In this work we have found a set of specific transcripts related to metabolism and the immune system that are differentially expressed during AHPND. This new information has been added and highlighted in the manuscript, mainly in the discussion section. |
| Hub transcripts are interesting, but discussion about it is not much | In the latest discussion we have included more information about hub transcripts generated from protein-protein networks. |
| The data from transcriptome sequencing did not support and explain effectively histopathological analysis | Histopathological data is now included, to demonstrate the presence of AHPND in hepatopancreas, and it also gives us additional information about the progress of the lesions. |
| Line 90, “In this context...”, but the following part is not the result of this study. | Correction, done. |
| Line 448, did “Interaction networks of DETs predicted by GeneMANIA” correspond to S1 Fig | Correction, done. |
| Writing should be improved (e.g. L162, L556, L562) | Corrections done. |
| **REVIEWER 3** | |
| **Comments to the Author** | **Answer to reviewer** |
| Please provide the adequate information on Vibrio parahaemolyticus IPNGS16 used in this study. Is the genome sequence  of this Vibrio strain available | The strain was recently isolated from a shrimp farm located in Guasave- Sinaloa, Mexico. There is not a genome sequence available for this strain yet, in L124 we described the available information that we have at this moment. |
| The LC50 of 660 CFU/ml is quite different than that report for other VPAHPND strains in the previous studies. Please  justified your results compared to other studies | Our results compared to Joshi et al., 2014 and López-León et al., 2016 are to a certain extent related, we have a *V. parahaemolyticus* IPNGS16 strain more aggressive to the one used in previous studies. Joshi et al., 2014 shows different AHPND strains with different pathogenic capacity. In addition, the organisms used in the experimental infection come from a genetic line (growth improved) different to López-León et al., 2016 (WSSV-resistant), which may increase its susceptibility to AHPND. |
| All sections were poorly written especially the Result Part. Why the authors wrote some descriptions similar to the Figure  legends in the Result Part? (For examples, Lines 312-315; Lines 328-332 etc.). Please remove the redundancy | Corrections done. |
| Please remove Table 1 and provide only necessary results to the readers. | We think that Table I is important, because it represents the MixS Standard, which is the minimum information that an RNA-seq study should have. For this reason we have leave it as a Supplementary Table (S1 Table). |
| I could not find the logic of choosing thrombospondin hub over the others | We chose thrombospondin because two authors found the gene up-regulated during the AHPND, in this study also presented. In addition, in the protein-protein analysis presented in this paper, we observe as hub gene part of the top-20. We would like to emphasize the thrombospondin gene response during AHPND. (Duan et al., 2016 & Soonthornchai *et al.,* 2016). |
| What could be concluded from the 10 genes that were selected for qRT-PCR validation? This also reflect the conclusion in  the Abstract on hemocyanin and clottable protein function in neutralizing the toxins. | Discussion and conclusion were re-written with a new focus on the 10 selected genes. |

**References**

López-León, P., Luna-González, A., Escamilla-Montes, R., Flores-Miranda, M. C., & Fierro-Coronado, J. A. (2016). Isolation and characterization of infectious Vibrio parahaemolyticus, the causative agent of AHPND , from the whiteleg shrimp ( Litopenaeus vannamei ). *Lat. Am. J. Aquat. Res.*, *44*(3), 470–479. <https://doi.org/10.3856/vol44-issue3-fulltext-5>

Joshi, J., Srisala, J., Truong, V. H., Chen, I. T., Nuangsaeng, B., Suthienkul, O., … Thitamadee, S. (2014). Variation in Vibrio parahaemolyticus isolates from a single Thai shrimp farm experiencing an outbreak of acute hepatopancreatic necrosis disease (AHPND). *Aquaculture*, *428*–*429*, 297–302. https://doi.org/10.1016/j.aquaculture.2014.03.030

Y. Duan, J. Li, Z. Zhang, J. Li, P. Liu, Characterization of ADP ribosylation factor 1 gene from Exopalaemon carinicauda and its immune response to pathogens challenge and ammonia-N stress, Fish. Shellfish Immunol. 55 (2016) 123-130.

Soonthornchai, W., Chaiyapechara, S., Klinbunga, S., Thongda, W., Tangphatsornruang, S., Yoocha, T., … Jiravanichpaisal, P. (2016). Differentially expressed transcripts in stomach of Penaeus monodon in response to AHPND infection. *Developmental and Comparative Immunology*, *65*, 53–63. https://doi.org/10.1016/j.dci.2016.06.013
