# Supplementary material for: Transcriptomic analysis of Pacific white shrimp (Litopenaeus vannamei, Boone 1931) in response to acute hepatopancreatic necrosis disease caused by Vibrio parahaemolyticus
Source: PLoS One. 2019 Aug 13;14(8):e0220993. doi: 10.1371/journal.pone.0220993 (PMC6692014; doi:10.1371/journal.pone.0220993)
Supplement: Attachment — Submitted filename: Response to Reviewers.docx [file pone.0220993.s011.docx]

La Paz, Baja California Sur, Mexico, July 23^th^ 2019

**Irene Söderhäll**

**Academic Editor**

**PLOS ONE**

We greatly appreciate the revision of our manuscript and thank the reviewers for their generous comments on the manuscript. We have edited the manuscript to address their concerns. In particular the comments from reviewer **#1** and comments related to PLOS ONE style. The response to the reviewers' concerns is detailed on the next page of the letter.

We believe that the manuscript is now suitable for publication in PLOS ONE journal.

Sincerely yours, on behalf of all authors

__________________________

**Dr. Felipe Ascencio**

ascencio@cibnor.mx

*Reviewer 1*

*Basic reporting*

*Reviewer #1: The manuscript reports the transcriptome analysis of the L. vannamei hepatopancreas during AHPND infection. A total of 915 differentially expressed transcript were found and categorized. Protein-protein network analysis was performed and hub genes were identified. Ten selected candidate genes were further analyzed by qRT-PCR to validate the RNA-seq differential expression data. The analysis data suggest a close interaction between the hepatopancreas metabolism and immune system, indicating their important during AHPND infection. The manuscript contains informative data that lead to the understanding of host immune response to AHPND infection. A few comments and suggestions are listed below”*

*1. Figure legends should be separated from the Results Part. Most figure legends should be rewritten to provide adequate information of the experiments as they must be self-explanatory. Some information such as number of shrimps, infection method, no. of repeated experiment should be included.*

*2. It is suggested that the gene name should be labeled in Figs. 8 and 9 so the readers could easily follow the gene expression of each selected gene. The x-axis should be changed to time post infection (hpi) to correspond to the text.*

*3. Please add to discussion the DE genes found in this study vs those reported in previous studies. What are the differences and new finding?*

*4. Line 336, CL50 should be changed to LC50*

1. Based on Plos one submission guidelines, the following is mentioned (in figures section): "Place figure captions in the manuscript text in read order, immediately following the paragraph where the figure is first cited Do not include captions as part of the file or submit them in a separate document" (Figures and Captions guidelines). For this reason the legends cannot be moved from the results side. The comment that the figures should be self-explanatory has been taken into account. The figures captions were restructured to make the figures easier to read.
2. Correction done (FIGS 8 and 9).
3. Information discussed in L683-708.
4. Correction done (L313).
